# Similar Epitope Specificities of IgG and IgA Antibodies Elicited by Ad26 Vector Prime, Env Protein Boost Immunizations in Rhesus Monkeys

**DOI:** 10.1128/JVI.00537-18

**Published:** 2018-07-17

**Authors:** Zi Han Kang, Christine A. Bricault, Erica N. Borducchi, Kathryn E. Stephenson, Michael S. Seaman, Maria Pau, Hanneke Schuitemaker, Danielle van Manen, Frank Wegmann, Dan H. Barouch

**Affiliations:** a Center for Virology and Vaccine Research, Beth Israel Deaconess Medical Center, Harvard Medical School, Boston, Massachusetts, USA; b Janssen Infectious Diseases and Vaccines, Leiden, Netherlands; c Ragon Institute of MGH, MIT and Harvard, Cambridge, Massachusetts, USA; Ulm University Medical Center

**Keywords:** IgA, IgG, human immunodeficiency virus, vaccines

## Abstract

Vaccine-elicited immunoglobulin G (IgG) has been shown to be important for protection against simian-human immunodeficiency virus (SHIV) infection in rhesus monkeys. However, it remains unclear whether vaccine-elicited IgA responses are beneficial or detrimental for protection. In this study, we evaluated the kinetics, magnitude, breadth, and linear epitope specificities of vaccine-elicited IgG and IgA responses in serum and mucosal secretions following intramuscular immunization with adenovirus 26 (Ad26) prime, Env protein boost vaccination regimens. The systemic and mucosal antibody responses exhibited kinetics similar to those of the serum antibody responses but lower titers than the serum antibody responses. Moreover, the IgG and IgA responses were correlated, both in terms of the magnitude of the responses and in terms of the antibody specificities against linear human immunodeficiency virus type 1 (HIV-1) Env, Gag, and Pol epitopes. These data suggest that IgG and IgA responses are highly coordinated in both peripheral blood and mucosal compartments following Ad26/Env vaccination in rhesus monkeys.

**IMPORTANCE** Vaccine-elicited IgG responses are important for protection against simian-human immunodeficiency virus (SHIV) infection in nonhuman primates. However, much less is known about the role and function of IgA, despite it being the predominant antibody in mucosal sites. There is debate as to whether HIV-1-specific IgA responses are beneficial or detrimental, since serum anti-Env IgA titers were shown to be inversely correlated with protection in the RV144 clinical trial. We thus assessed vaccine-elicited IgG and IgA antibody responses in peripheral blood and mucosal secretions following vaccination with the Ad26/Env vaccine.

## INTRODUCTION

Human immunodeficiency virus type 1 (HIV-1) infection in humans is mainly transmitted via the mucosal route (1). It is therefore likely that a prophylactic vaccine will need to elicit protective antibody responses at the mucosal sites of infection (2). However, mucosal IgG and IgA responses following vaccination remain poorly characterized. Cervicovaginal secretions generally contain higher levels of IgG than IgA, whereas gastrointestinal secretions and saliva typically contain more IgA (3, 4).

The RV144 vaccine trial demonstrated 31.2% efficacy in preventing HIV-1 infection (5). Follow-up studies showed that while plasma IgG directed against the variable loop 1 and 2 (V1/V2) region in Env correlated directly with protection, plasma IgA binding to Env inversely correlated with protection (6). It has been hypothesized that IgA directed against the C1 region of Env may have interfered with the antibody-dependent cellular cytotoxicity effector (ADCC) function of the protective IgG responses (7).

However, HIV-specific IgA responses have also been previously linked to protection in certain models. In HIV-1-exposed, persistently seronegative individuals, HIV-specific IgA antibodies were detected in both the serum and mucosal secretions (8–11), and mucosal and plasma IgA purified from these seronegative individuals inhibited HIV mucosal transcytosis *in vitro* (8, 9). In rhesus macaques that were passively immunized with IgA1, IgA2, and IgG1 versions of a neutralizing monoclonal human antibody, HGN194, IgA1 provided the best protection against a simian-human immunodeficiency virus (SHIV) challenge, and only IgA1 blocked the transcytosis of cell-free virus across the epithelial layer *in vitro*, even though all three versions had similar neutralizing activities (12).

Thus, it remains unclear whether vaccine-induced peripheral and mucosal IgA responses are beneficial or detrimental for protection against infection. The adenovirus 26 (Ad26) prime, Env protein boost (Ad26/Env) vaccine has previously been shown to provide partial protection against simian immunodeficiency virus mac251 (SIVmac251) and SHIV-SF162P3 challenges (13), and this vaccine has recently been advanced into a phase 2b proof-of-concept study in humans (HVTN705, HPX2008). In this study, we evaluated the magnitude and epitope specificity of IgG and IgA elicited by Ad26/Env vaccination in rhesus monkeys.

## RESULTS

### Vaccination regimens.

We utilized a study originally designed to test five different adenovirus 26 (Ad26) vector and Env protein immunization regimens (Table 1) for a detailed evaluation of IgG and IgA antibody responses. Twenty rhesus macaques (Macaca mulatta) were primed intramuscularly (i.m.) with either a 3- or 4-valent Ad26 vector regimen at weeks 0 and 12 and then were boosted by Env gp140 protein at weeks 24 and 48, either alone or in combination with Ad26 vectors. Two groups (*n* = 8) also received an additional Env gp140 boost at week 76, whereas three groups (*n* = 12) did not receive an Env gp140 boost at week 76.

**TABLE 1:**
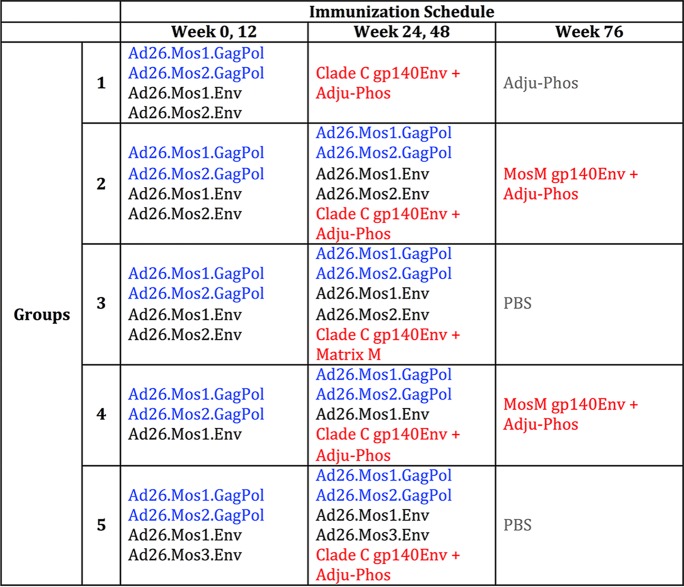
Immunization regimens of rhesus macaques^*a*^

a A total of 1 × 10^10^ viral particles (v.p.) of Ad26 vectors, 250 μg of C97ZA012 gp140 (clade C; C97) envelope protein, 60 μg of the adjuvant Matrix M, or 850 μg of the adjuvant Adju-Phos was administered intramuscularly (i.m.) in the quadriceps of rhesus macaques (*n* = 4 per group), as detailed in the table. Animals were immunized on weeks 0, 12, 24, 48, and 76. Each Ad26 vector carries mosaic Gag-Pol transgene sequences and mosaic Env transgene sequences. These mosaic sequences have been bioinformatically designed to optimize cellular immunologic coverage of the global HIV-1 sequence diversity (39, 40). Text colors indicate different vaccination regimens: blue, Ad vectors with a Gag-Pol insert; black, Ad vectors with an Env insert; red, Env protein and adjuvant; gray, adjuvant alone or PBS.

**FIG 1:**
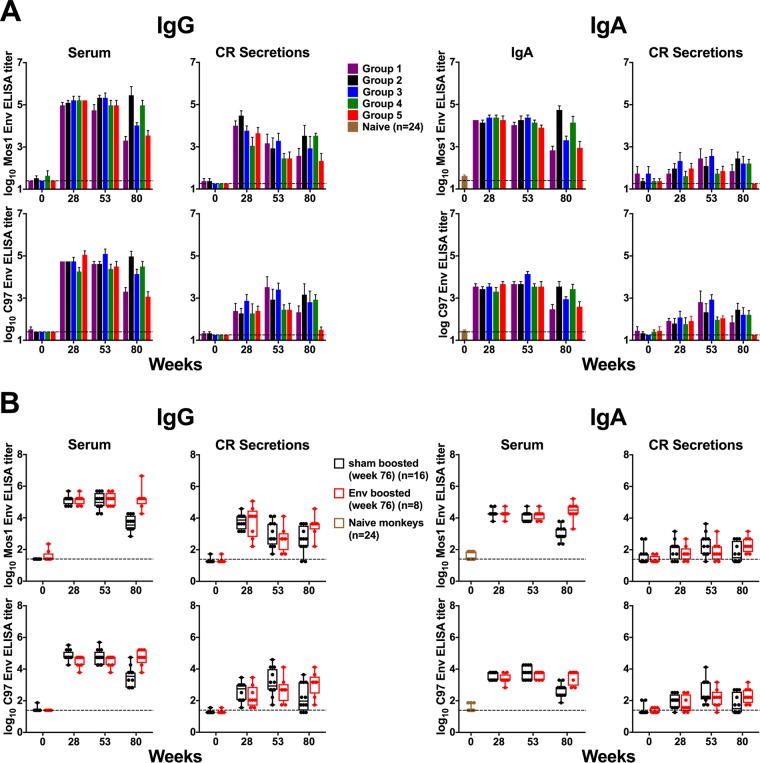
Envelope-specific IgG and IgA antibody responses elicited following i.m. vaccination in serum and colorectal secretions. Rhesus monkeys were immunized i.m. with either a 3- or 4-valent Ad26 prime-boost regimen, as detailed in Table 1, on weeks 0, 12, 24, and 48. On week 76, animals were subsequently either sham boosted or boosted with a clade C envelope protein (C97ZA012 gp140). Serum and colorectal (CR) samples were assessed for Env-specific IgG and IgA binding antibody responses by endpoint ELISA, using clade C (C97) and mosaic (Mos1) Env coating proteins at weeks 0, 28, 53, 76, and 80. Horizontal broken lines represent the assay threshold. Serum samples from 24 naive monkeys were used to establish a baseline for Env-specific IgA responses in serum. (A) Antibody responses are shown for individual immunization regimen groups. Mean and standard error of mean (SEM) values are shown. (B) Antibody responses for animals that did (*n* = 8) or did not (*n* = 12) receive the week 76 Env protein boost were pooled. The median for the endpoint ELISA titers is shown.

These regimens all induced a similar magnitude of Env-specific binding antibodies by enzyme-linked immunosorbent assay (ELISA) at weeks 28 and 53 (Fig. 1A). To evaluate vaccine-induced IgG and IgA responses, we pooled the results for animals that either did (*n* = 8) or did not (*n* = 12) receive the week 76 Env gp140 boost for subsequent analyses. Animals within these two groups that received different regimens shared similar magnitudes and kinetics of binding antibody responses (Fig. 1A).

### Serum and mucosal binding antibody titers are correlated following vaccination.

To assess Env-specific binding antibody titers from serum and colorectal secretions, we assessed samples from 4 weeks after each Env immunization (weeks 28, 53, 80) by ELISA. Consistent with our previous observations (14), Env-specific IgG and IgA responses were elicited in both the serum and mucosal compartments, and the kinetics of the mucosal antibody titers closely mimicked those in serum (Fig. 1A and B). However, mucosal antibody titers were 1.5 to 2.0 logs lower than those found in serum (median, 2.051 logs for Mos1 and 1.574 logs for C97), and Env-specific IgA titers were 0.5 to 1.0 log lower than Env-specific IgG titers (median, 0.953 log for serum responses and 0.477 log for mucosal responses). Antibody titers at week 80 were higher in the groups that received the additional week 76 boost than in the groups that did not receive the week 76 boost (*P* < 0.005 for all serum responses; *P* < 0.05 for mucosal responses, except for the C97-specific IgG mucosal response [*P* = 0.0516]) (Fig. 1B).

**FIG 2:**
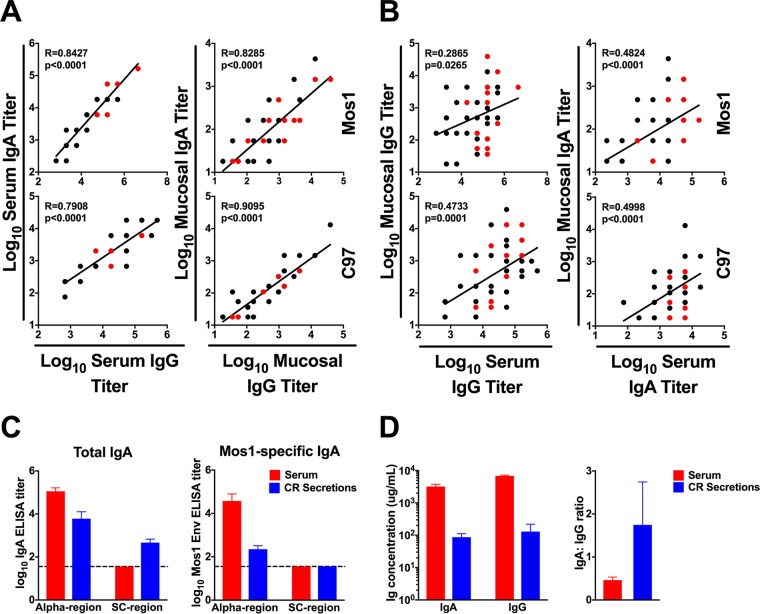
Analysis of vaccine-elicited Env-specific IgG and IgA binding antibody responses in serum and colorectal secretions. (A and B) Correlations between C97 or Mos1 Env-specific IgG and IgA binding antibody titers in serum and colorectal samples were evaluated for weeks 28, 53, and 80. (A) Correlations between C97 or Mos1 Env-specific IgG and IgA responses in serum (left) and mucosal secretions (right). (B) Correlations between C97 or Mos1 Env-specific serum and mucosal antibody titers for IgG (left) and IgA (right). Correlations were analyzed using Spearman rank-correlation tests. (C and D) Week 80 serum and colorectal secretion samples from monkeys boosted 4 weeks earlier with the Env protein (*n* = 3) were then evaluated for the presence of the secretory component (SC) region in total and Mos1 Env-specific IgA and total IgG and IgA concentrations (C) and the IgA/IgG ratio (D). Mean and SEM values are shown.

IgG versus IgA antibody titers were tightly correlated in both serum and mucosal compartments (for Mos1, *r* = 0.8427 for the serum compartment and *r* = 0.8285 for the mucosal compartment; for C97, *r* = 0.7908 for the serum compartment and *r* = 0.9095 for the mucosal compartment; *P* < 0.0001, Spearman rank-correlation test) (Fig. 2A). Serum IgG versus mucosal IgG antibody titers were also significantly correlated (*r* = 0.2865 and *P* = 0.0265 for Mos1-specific responses; *r* = 0.4733 and *P* = 0.0001 for C97-specific responses), as were serum IgA versus mucosal IgA antibody titers (*r* = 0.4824 and *P* < 0.0001 for Mos1; *r* = 0.4998 and *P* < 0.0001 for C97) (Fig. 2B). These data extend previous findings showing that Env-specific mucosal antibody responses correlated with the systemic antibody responses following vaccination (15). This correlation was observed at multiple time points (data not shown), suggesting that the responses were immunologically coordinated.

To investigate whether the Env-specific IgA in colorectal secretions represented transudation of serum IgA into the colorectal mucosa, we assessed mucosal Mos1-specific IgA for the presence of the IgA-containing secretory component region (SC region), which is present on secretory IgA in mucosal secretions (16). There was no detectable SC region in Mos1-specific IgA in colorectal samples (Fig. 2C). However, total IgA containing the SC region was readily detected in colorectal secretions, while no SC region-specific IgA was detected in serum, as expected (Fig. 2D). The IgA/IgG ratio was different in the serum and mucosal compartments, suggesting that there was little to no contamination of these mucosal samples by serum. These data are consistent with a model in which the majority of Env-specific IgA in mucosal secretions reflects transudation of serum IgA rather than locally produced IgA.

### NAb titers are correlated with binding antibody titers following vaccination.

**FIG 3:**
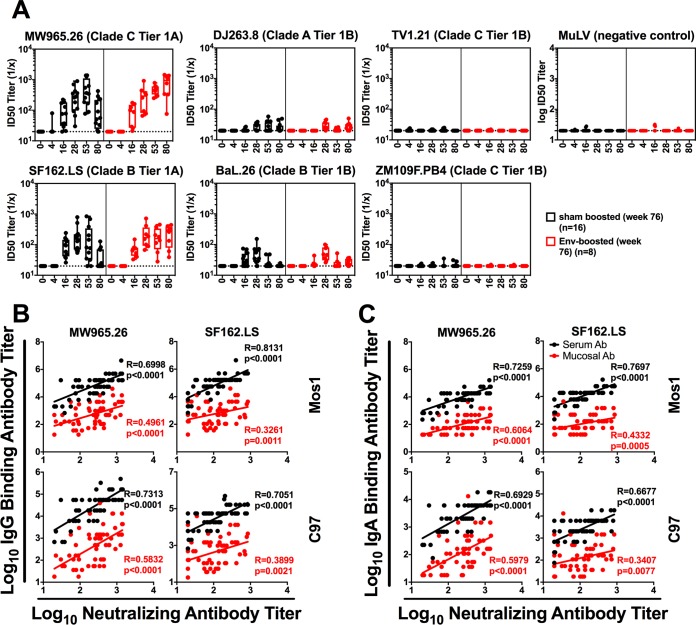
Peripheral neutralizing antibody responses elicited by systemic i.m. immunizations. Rhesus monkeys were immunized i.m. with either a 3- or 4-valent Ad26 prime-boost regimen, as detailed in Table 1, on weeks 0, 12, 24, and 48. On week 76, animals were subsequently either sham boosted (*n* = 12) or boosted with a clade C envelope protein (*n* = 8). (A) Serum neutralizing antibody responses were analyzed across a panel of tier 1A (MW965.26 and SF162.LS) and tier 1B (DJ263.8, BaL.26, TV1.21, ZM109F.PB4) pseudoviruses, and the 50% infective dose (ID_50_) was determined. Murine leukemia virus (MuLV) was used as a negative control. Horizontal broken lines represent the assay threshold. The median for neutralizing endpoint titers is shown. (B and C) Correlations between Mos1 Env-specific and C97 Env-specific binding IgG (B) and IgA (C) antibody responses in either serum (black) or mucosal (red) samples and neutralizing antibody (Ab) responses to MW965.26 and SF162.LS pseudoviruses for weeks 28, 53, and 80 after the first immunization. Correlations were analyzed using Spearman rank-correlation tests.

To determine the neutralizing capacity of antibodies elicited by the different immunization regimens, neutralizing antibody (NAb) responses from serum samples were analyzed across a panel of tier 1A (highly sensitive) and tier 1B (less sensitive) pseudoviruses using the TZM.bl neutralization assay (17, 18). As previously reported, these vaccines do not induce tier 2 NAb responses (13). Higher neutralizing antibody titers were elicited against the tier 1A pseudoviruses (MW965.26 and SF162.LS) than against the tier 1B pseudoviruses (DJ263.8 and BaL.26), and neutralizing antibody responses generally increased in magnitude following each immunization (Fig. 3A). At week 80, only those animals that were boosted at week 76 (red bars) showed an increase in the magnitude of their neutralizing antibody titers, as expected (Fig. 3A). We were unable to assess neutralizing antibody responses in colorectal secretions due to insufficient sample volume. The C97 and Mos1 Env-specific mucosal and serum binding IgG and IgA titers correlated with the tier 1A neutralizing antibody responses (Fig. 3B and C, respectively; *r* > 0.65 and *P* < 0.0001 for all IgG correlations; *r* > 0.34 and *P* < 0.005 for all IgA correlations; Spearman rank-correlation tests). Weaker correlations between binding antibody titers and neutralizing antibody titers were observed against tier 1B pseudoviruses (data not shown), likely due to the low tier 1B responses.

### IgG and IgA elicited by vaccination have similar linear antibody epitope breadth and depth.

**FIG 4:**
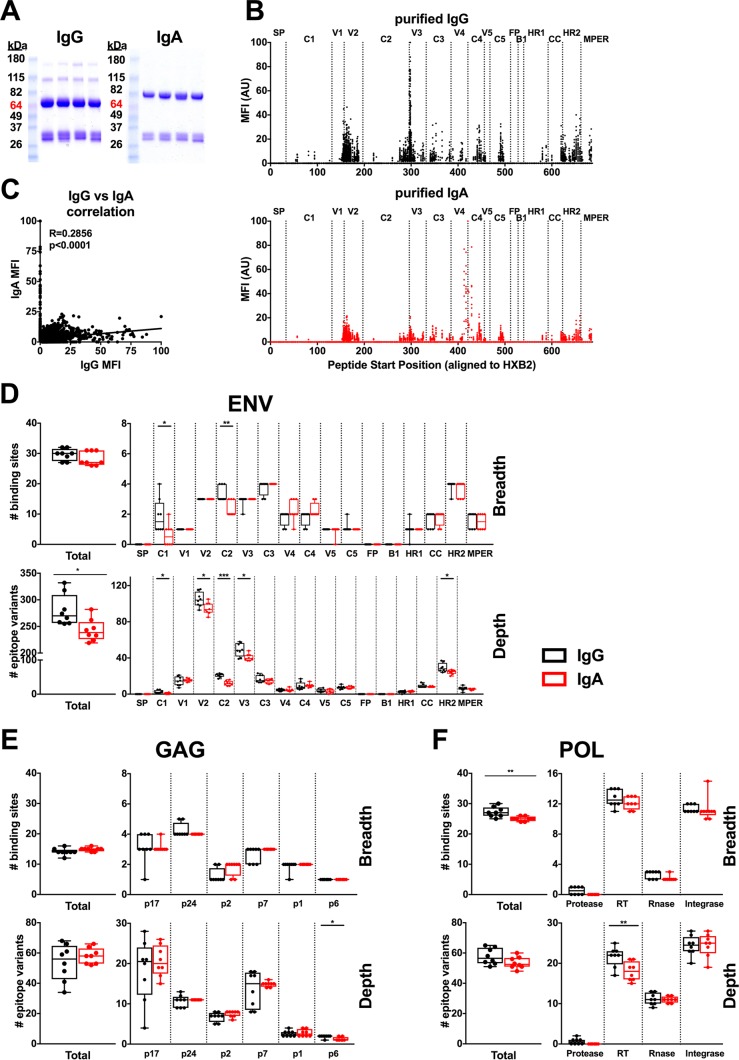
Purified IgG and IgA binding to linear Env, Gag, and Pol peptides by peptide microarray. IgG and IgA binding to Env, Gag, or Pol linear 15-mer peptides was analyzed by peptide microarray, using purified IgG and IgA from week 80 serum samples from monkeys boosted 4 weeks earlier with a clade C Env protein (C97ZA012 gp140) (groups 2 and 4, *n* = 8). (A) Representative Coomassie-stained SDS-PAGE gels of purified IgG and IgA. Each lane represents purified IgG or IgA antibody from an individual animal. (B) The normalized mean fluorescence intensity (MFI) of the IgG (black, top) and IgA (red, bottom) responses against Env linear peptides is plotted against the Env peptide start position (HXB2 numbering). MFI values for the IgG or IgA responses for each animal were normalized to a scale of 100 arbitrary units (AU), based on the highest MFI value for each monkey. The MFI values for all 8 monkeys are depicted. (C) Correlation between IgG and IgA normalized MFI values for Env peptides. Correlations were analyzed using Spearman rank-correlation tests. (D to F) The breadth (the number of binding sites per region) and depth (the number of epitope variants per region) for each immunized monkey are plotted for purified IgG and IgA responses against Env (D), Gag (E), and Pol (F) linear peptides. The breadth and depth of the antibody responses against peptides from the entire Gag, Pol, or Env protein (left) or individual regions within each protein (right) are both depicted. Horizontal bars depict the median for breadth or depth. Statistical significance was analyzed using the Mann-Whitney test (*, *P* ≤ 0.05; **, *P* ≤ 0.01; ***, *P* ≤ 0.001). RT, reverse transcriptase.

IgG and IgA were separately purified from week 80 serum samples using a protein G-agarose column (IgG) and a peptide M-agarose column (IgA), respectively (19). An SDS-PAGE gel was run to determine the purity of the samples (Fig. 4A). Purified IgG showed reactivity similar to that seen on ELISAs (data not shown). To determine the diversity of the linear epitopes targeted by vaccine-elicited IgG or IgA, purified IgG and IgA samples from week 80 serum were assessed by peptide microarrays containing 6,564 HIV-1 15-amino-acid-long peptides, as previously described (20), using the groups that were boosted at week 76 and that had the highest binding and neutralizing antibody responses.

Purified serum IgG and IgA linear binding antibody responses were analyzed for Env, Gag, and Pol. The pattern of IgG and IgA binding against linear Env peptides was generally similar, with both IgG and IgA responses predominantly being directed against the V1/V2, V3, C4, C5, and HR2 epitopes, although there was more IgG binding to V3 epitopes and more IgA binding to C4 epitopes (Fig. 4B). Only minimal IgA responses were directed to C1, which has been reported to be a target for IgA that interferes with IgG-mediated ADCC activity (7). There was also a correlation between the normalized mean fluorescent intensity (MFI) of IgG and that of IgA (*r* = 0.2856; *P* < 0.0001, Spearman rank-correlation test) (Fig. 4C), consistent with the ELISA data (Fig. 2A). The breadth and depth of IgG binding to linear Env, Gag, and Pol peptides were similar to the breadth and depth of IgA binding to these regions (Fig. 4D to F). IgG trended toward a slightly greater breadth of peptide binding than IgA, and its depth against Env linear epitopes was slightly higher than that of IgA (*P* = 0.011) (Fig. 4D), but overall, IgG and IgA showed comparable profiles.

For individual Env regions, we similarly observed a trend of IgG recognizing a slightly greater breadth and depth of epitopes than IgA, particularly in the C1 and C2 regions for breadth and in the C1, C2, V2, V3, and HR2 regions for depth (Fig. 4D). However, these differences were sporadic and modest and likely reflect the overall higher magnitude of IgG responses. Similarly, we observed similar IgG and IgA breadths and depths of antibody responses within individual Gag (Fig. 4E) and Pol (Fig. 4F) regions. The protease region in Pol was not included in the Ad vectors, and thus, no binding was observed for protease epitopes, as expected (Fig. 4F).

### IgG and IgA from vaccinated monkeys generally target similar linear epitopes.

**FIG 5:**
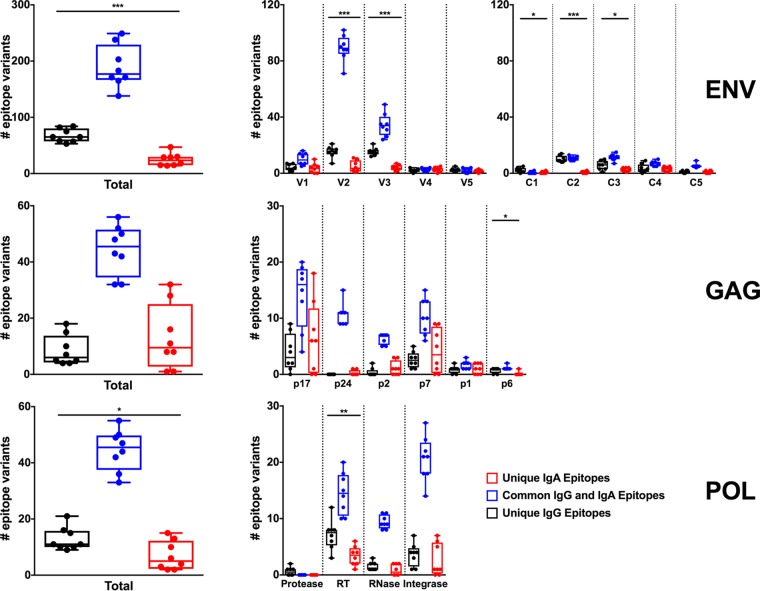
Comparison of IgG and IgA linear epitope binding. Env (V1 to V5 and C1 to C5 regions only), Gag, and Pol linear epitopes targeted by purified IgA and IgG from serum were compared for sequence similarity. Blue bars depict common sequences that are targeted by both IgA and IgG responses, while the black or red bars depict sequences that are uniquely targeted by either IgG or IgA, respectively. Horizontal bars represent the median number of targeted sequences. Sequences were compared for individual regions within Env, Gag, and Pol (right), as well as for each protein in general (left). Only the V1 to V5 and C1 to C5 regions of Env were analyzed. Statistical significance was analyzed using the Mann-Whitney test (*, *P* ≤ 0.05; **, *P* ≤ 0.01; ***, *P* ≤ 0.001).

We next explored the extent to which IgG and IgA targeted similar linear epitopes in Env, Gag, and Pol. The IgG and IgA responses appeared to recognize similar linear Env, Gag, and Pol peptides, with the majority of epitopes being targeted by both IgG and IgA (Fig. 5) (mean, 67.1% for Env, 67.33% for Gag, 69.5% for Pol). However, there was a minority of epitopes that were targeted uniquely by either IgG (mean, 19.72% for Env, 12.91% for Gag, 19.92% for Pol) or IgA (mean, 4.31% for Env, 19.76% for Gag, 10.57% for Pol). Unique epitopes are defined as peptide sequences that differ by at least 1 amino acid. They are found across the antigens in regions of high epitope density common to both IgG and IgA. There were slightly more unique linear Env (*P* = 0.002) and Pol (*P* = 0.0393) epitopes targeted by IgG than by IgA (Fig. 5). This finding is consistent with the observation that the breadth and depth of IgG responses were slightly higher than those of IgA responses (Fig. 4D to F).

### Purified IgG from vaccinated monkeys only weakly targets the A32 epitope.

**FIG 6:**
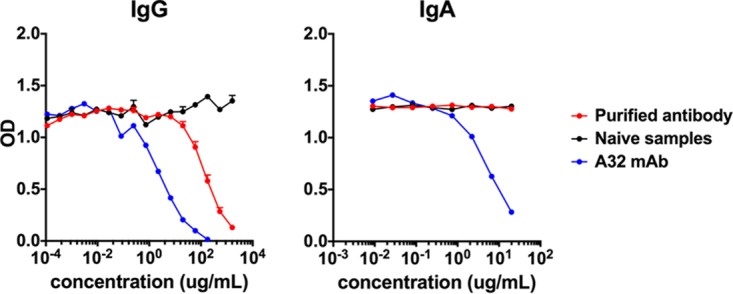
Competition ELISA with the A32 MAb. Purified IgG and IgA (*n* = 8) from week 80 serum samples from monkeys boosted 4 weeks earlier with a clade C Env protein (C97ZA012 gp140) were used in a competition ELISA to block binding of the A32 MAb to Mos1 Env. The A32 MAb was used as a positive control, and purified IgG and IgA from naive monkeys (*n* = 3) were used as a negative control. The mean and standard error of the mean are shown.

To determine whether purified IgA samples from this study bound the C1 conformational epitope that was previously found to interfere with the ADCC function of IgG (7), a competition ELISA was performed using the monoclonal antibody (MAb) A32. A32 binds a C1 conformational epitope (21) and was previously used to identify the C1 binding epitope of IgG-mediated ADCC in the RV144 trial (22). Using a competition ELISA against the Mos1 antigen, high concentrations of purified IgG (>6.67 μg/ml) competed with A32 binding. Purified IgA did not compete against A32 binding up to a concentration of 20 μg/ml (Fig. 6), but we were unable to use higher concentrations of IgA due to sample limitations. These data demonstrate that the IgA responses induced by the Ad26/Env vaccine did not compete with A32 binding at low concentrations.

## DISCUSSION

Mucosal surfaces represent the critical portals for HIV-1 entry, and thus, it is likely that mucosal antibody responses will be required by a prophylactic HIV-1 vaccine. While immunization via a mucosal route is optimal to generate mucosal antibody responses in certain models, it is also clear that parenteral immunizations can effectively induce both systemic and mucosal immune responses in animal models (23–25) and in humans (26–28).

In this study, we showed that intramuscularly administered Ad26/Env vaccines induced both serum and mucosal Env-specific IgG and IgA antibodies in rhesus macaques. The kinetics of the mucosal antibody responses were similar to those of the serum responses. Moreover, IgG and IgA responses were correlated in both anatomic compartments (Fig. 1 and 2A). These data suggest that systemic and mucosal antibody responses are immunologically coordinated, with mucosal antibodies likely reflecting transudation of serum antibodies into mucosal compartments (14, 29, 30). These findings confirm and extend prior studies. In monkeys that were systemically injected with monomeric or dimeric IgA forms of the broadly neutralizing antibody b12, both forms of IgA were found in mucosal compartments (30). Bound polymeric immunoglobulin receptor (pIgR) is subsequently cleaved to form the secretory component (SC). It is possible that the Env-specific IgA found in colorectal secretions of our vaccinated monkeys is transudated from serum, given the absence of SC region-containing Env-specific IgA (Fig. 2C).

IgA is the major antibody isotype present in mucosal secretions (1) and is important in protective responses against viral infections. However, it remains unclear whether HIV-1-specific IgA responses are beneficial or detrimental (31). Serum anti-Env IgA titers were directly correlated with acquisition risk in the RV144 clinical trial (6, 7), and the mechanism of this effect has been hypothesized to involve C1-specific IgA that reduces the ADCC function of Env-specific IgG (7). However, it has also been suggested that Env-specific HIV-1 IgA responses may only be markers and not actual mechanisms of risk of infection (32). Moreover, it is unclear whether correlates from an ALVAC/gp120 vaccine would be relevant for an Ad26/gp140 vaccine. We utilized the high-throughput peptide microarray as a tool to study vaccine-elicited IgA antibody responses in greater detail, with the limitation that conformational epitopes were not evaluated in this assay. Peptide microarrays were utilized to assess antibody diversity against HIV-1 linear epitopes (6, 33). We detected consistent systemic and mucosal IgA responses, which generally had lower titers than IgG responses. Purified IgG and IgA samples from our vaccinated monkeys bound to similar regions within the Env protein, predominantly in the V1 to V3, C4, and C5 regions (Fig. 4B), with minimal to no linear IgG and IgA responses being directed against the C1 linear epitope. The MAb A32 blocks ADCC and binds a conformational C1 epitope (22, 34). IgA did not block A32 binding at concentrations up to 20 μg/ml (Fig. 6).

Class switch recombination is mediated by activation-induced cytidine deaminase and results in the expression of one of the downstream isotypes (IgA, IgG, or IgE) from the expression of IgM or IgD on naive B cells. IgA is formed by class switching from either IgM or IgG intermediaries (35, 36), and it has been shown *in vitro* that IgA is preferentially formed by sequential switching through IgG intermediaries (37). Our results showing that IgG and IgA targeted similar Env, Gag, and Pol linear peptide sequences (Fig. 5) suggest that vaccine-elicited IgA and IgG may have common B cell precursors, and it is possible that IgA could have been elicited via class switching through an IgG intermediary. As IgG and IgA class switching is regulated by the local microenvironment, such as cytokines (38), these data raise interesting questions about how and when class switching occurs following immunization and how this may affect protection against subsequent infection.

Our findings demonstrate that the IgG and IgA responses in peripheral blood and colorectal secretions are tightly correlated following Ad26/Env vaccination, both in terms of the overall magnitude and in terms of the individual epitopes targeted. The Ad26/Env vaccine is currently being evaluated in a phase 2b clinical efficacy trial, and thus, whether vaccine-elicited IgA contributes to or detracts from protective efficacy is an important question that warrants further evaluation.

## MATERIALS AND METHODS

### Animals and immunizations.

Twenty adult rhesus monkeys (Macaca mulatta) were housed in the Alphagenesis Inc. Animal Research Facility. All studies were approved by the Alphagenesis Inc. Institutional Animal Care and Use Committee (IACUC). Priming immunizations at weeks 0 and 12 involved i.m. injections of 1 × 10^10^ viral particles (v.p.) of a nonreplicating recombinant adenovirus 26 (Ad26) vector expressing HIV mosaic Gag, Pol, and Env immunogens (Ad26.Gag-Pol and Ad26.Env) (39, 40). Animals were subsequently boosted i.m. at weeks 24 and 48 with either clade C gp140Fd trimer protein immunogen alone (250 μg/animal) or the combination of Ad26.Gag-Pol and Ad26.Env (1 × 10^10^ v.p./animal). Clade C gp140 trimer protein immunizations were adjuvanted with either Adju-Phos (850 μg) or Matrix M (60 μg). The antigens were formulated in a dose volume of 500 μl and administered via i.m. injections in the quadriceps muscles. The immunization regimens are detailed in Table 1.

### Antibody purification. (i) IgG purification.

IgG was purified from serum using a protein G-agarose column (2 ml of protein G-agarose beads). The columns were washed with protein G IgG binding buffer (catalog number 21019; Thermo Scientific), and bound IgG was subsequently eluted with 0.1 M glycine (pH 2 to 3) and immediately neutralized with 1 M Tris (pH 8). The different IgG eluate fractions were then pooled and buffer exchanged with 1× phosphate-buffered saline (PBS) using an Amicon Ultra 10K device spin column.

### (ii) IgA purification.

IgA was purified from IgG-depleted serum with a peptide M-agarose column (2 ml of peptide M-agarose beads). Columns were washed with peptide M IgA binding buffer (10 mM sodium phosphate, 150 mM sodium chloride, pH 7.2), and bound IgA was subsequently eluted with 0.1 M glycine (pH 2 to 3) and immediately neutralized with 1 M Tris (pH 8). The different IgA eluate fractions were then pooled, run over a protein G-agarose column again to remove any contaminating IgG, and subsequently buffer exchanged with 1× PBS using an Amicon Ultra 10K device spin column.

### ELISA. (i) Serum IgG and IgA.

Serum binding antibody titers against HIV-1 Env were determined by endpoint ELISAs as previously described (41). Briefly, 96-well MaxiSorp ELISA plates (Thermo Fisher Scientific) were coated overnight with 100 μl per well of HIV-1 Env at a concentration of 1 μg/ml in PBS and subsequently blocked for 4 h with PBS containing 2% bovine serum albumin (BSA; Sigma) and 0.05% Tween 20 (Sigma). Serum was serially diluted and incubated for 1 h at room temperature. The plates were washed 3 times with PBS containing 0.05% Tween 20 and were incubated for 1 h with horseradish peroxidase (HRP)-conjugated anti-IgG (Jackson ImmunoResearch Laboratories Inc.) or biotin-conjugated anti-IgA (Alpha Diagnostic International). Plates incubated with biotin-conjugated anti-IgA were washed 3 times and subsequently incubated with streptavidin-HRP. The plates were washed 3 times and developed with SureBlue tetramethylbenzidine microwell peroxidase (KPL Research Products); the reaction was stopped by the addition of stop solution (KPL Research Products), and the plates were analyzed at 450 nm/550 nm on a SpectraMax Plus ELISA plate reader (Molecular Devices) using Softmax Pro-4.7.1 software.

Total IgG and IgA concentrations were determined using a kit from Alpha Diagnostic International.

### (ii) Mucosal IgG and IgA.

For monkey mucosal secretion ELISAs, plates were instead incubated with a biotin-conjugated secondary antibody (Alpha Diagnostic International) for 2.5 h at 37°C, washed 3 times, and incubated with streptavidin-HRP for 1 h before developing. ELISA endpoint titers were defined as the highest reciprocal serum or mucosal secretion dilution that yielded an absorbance greater than 2-fold the background (IgG responses) or 3-fold the background (IgA responses).

### Neutralizing antibody assay in TZM.bl cells.

Neutralizing antibody responses against HIV-1 Env pseudovirions were measured using luciferase-based virus neutralization assays in TZM.bl cells (17, 18, 41, 42). These assays measure the reduction in luciferase reporter gene expression in TZM.bl cells following a single round of virus infection. The 50% infective dose (ID_50_) was calculated as the serum dilution that resulted in a 50% reduction in relative luminescence units compared with that for the virus control wells after the subtraction of cell control relative luminescence units. Threefold serial dilutions of serum samples were performed in duplicate (96-well flat-bottomed plate) in 10% Dulbecco modified Eagle medium (DMEM) growth medium (100 μl per well). Virus was then added to each well, and the plates were incubated for 1 h at 37°C. TZM.bl cells were then added (1 × 10^4^ per well in a 100-μl volume) in 10% DMEM growth medium containing DEAE-dextran (Sigma) at a final concentration of 11 μg/ml. Murine leukemia virus (MuLV) was used as a negative control in all assays. HIV-1 Env pseudoviruses, including clade A (DJ263.8), clade B (SF162.LS and BaL.26), and clade C (MW965.26) isolates, were prepared as previously described (42).

### Peptide microarrays.

Microarray slides were incubated with purified antibody (20). Purified IgG (1 μg/ml) and purified IgA (5 μg/ml) were diluted 1/10 in SuperBlock T20 (Tris-buffered saline [TBS]) blocking buffer (Thermo Scientific) and incubated for 1 h at 30°C with the peptide microarray slide. The slides were then washed with 5 ml of TBS buffer–0.1% Tween 20 for 3 min on a shaker at room temperature for 5 washes. Next, the slides were placed in the individual chambers of a Sarstedt Quadriperm dish and incubated with Alexa Fluor 647-conjugated AffiniPure mouse anti-human IgG (H+L) (Jackson ImmunoResearch Laboratories Inc.) and biotin-conjugated anti-monkey IgA (Alpha Diagnostics International) for 1 h at room temperature. The slides were then washed 5 times with TBS buffer–0.1% Tween 20. Cy3-conjugated streptavidin (Jackson ImmunoResearch Laboratories Inc) was added to the slide, and the slide was incubated in the dark for 1 h at room temperature. The slides were then washed 5 times with TBS buffer–0.1% Tween 20 and 5 times with deionized water. To dry, the slides were placed in a 50-ml Falcon tube and spun at 1,400 rpm for 5 min. A control slide incubated with secondary antibodies alone without sample was also run to determine the background.

### Microarray image analysis.

Slides were scanned with a GenePix 4300A scanner (Molecular Devices), using 635-nm and 532-nm lasers at 500 photomultiplier tube (PMT) and 100-power settings. Images were saved as TIF files. The fluorescent intensity for each feature (peptide spot) was calculated using GenePix Pro 7 software and a GenePix Array List (GAL) file. We then calculated the mean fluorescent intensity across the triplicate subarrays using a custom-designed R script and R software package 2.15.2. The threshold value used to define a minimum positive fluorescent intensity was calculated for each slide using the computational tool rapmad and a custom-designed R script. Data from each individual slide were combined with data from the control slide to create two distributions of data (noise and signal). The threshold values for positivity were defined as 5 standard deviations above the mean of the noise distribution (SD.noise × 5). As different fluorophores and lasers were used to detect IgG and IgA, the mean fluorescent intensity (MFI) values of reactive peptides for each monkey were normalized to a scale of 100.

An antibody epitope was defined to be 5 to 15 amino acids long (with the minimum epitope for antibody binding being 5 amino acids long), the breadth was defined to be the number of amino acid regions within any given HIV-1 protein (e.g., Env, Gag, Pol) region (e.g., V1 and V2 for HIV-1 Env) spanning an 11-amino-acid stretch. We defined the depth to be the number of unique sequences within an overlapping region of 5 to 15 amino acids (20).

### Comparison of IgG versus IgA epitopes.

To compare the common versus unique linear epitopes targeted by IgG and IgA, we compared the lists of reactive peptide sequences targeted by both IgG and IgA (or both) within each animal. The reactive peptide sequences for IgG and IgA in the same animal were aligned against each other to eliminate overlap. If any reactive peptide sequences shared 5 or more amino acids, we conservatively assumed that the peptides reflected the same epitopes. If the first and last overlapping peptide in a string of overlapping peptides shared 4 or fewer amino acids, we assumed that the peptides were recognized by a minimum of two antibody-binding sites. The list of unique linear epitope sequences for IgG was then compared to the list of unique sequences for IgA to determine which sequences were common (versus unique) to both IgG and IgA.

### Competition ELISA.

Ninety-six-well MaxiSorp plates were coated overnight with 1 μg/ml of Mos1 (100 μl per well). They were washed with PBS containing 0.05% Tween 20 and subsequently blocked for 3 h with PBS containing 1% BSA and 0.05% Tween 20. Purified serum IgG and IgA were then added in serial dilutions (with a starting concentration of 1,620 μg/ml and 20 μg/ml, respectively) and incubated for 1 h at room temperature. The plates were washed and incubated for 1 h with biotinylated A32 (biotinylation kit, EX-Link Micro NHS-PEG4-Biotinylation). The amount of A32-biotin added was previously determined by choosing a concentration of A32-biotin with 0.28 μg/ml of streptavidin-HRP that gave an optical density (OD) of about 1. The plates were then washed and incubated with 0.28 μg/ml of streptavidin-HRP for an hour and developed with SureBlue tetramethylbenzidine microwell peroxidase (KPL Research Products); the reaction was stopped by the addition of stop solution (KPL Research Products), and the plates were analyzed at 450 nm/550 nm on a SpectraMax Plus ELISA plate reader (Molecular Devices) using Softmax Pro-4.7.1 software.
